# An analysis of the use of targeted therapies in patients with advanced Merkel cell carcinoma and an evaluation of genomic correlates of response

**DOI:** 10.1002/cam4.4138

**Published:** 2021-07-16

**Authors:** Todd C. Knepper, Robyn A. Panchaud, Elnara Muradova, Leah Cohen, James A. DeCaprio, Nikhil I. Khushalani, Kenneth Y. Tsai, Andrew S. Brohl

**Affiliations:** ^1^ Department of Individualized Cancer Management H. Lee Moffitt Cancer Center and Research Institute Tampa FL USA; ^2^ Department of Anatomic Pathology H. Lee Moffitt Cancer Center and Research Institute Tampa FL USA; ^3^ Department of Medical Oncology Dana‐Farber Cancer Institute Boston MA USA; ^4^ Department of Medicine Brigham and Women’s Hospital and Harvard Medical School Boston MA USA; ^5^ Department of Cutaneous Oncology H. Lee Moffitt Cancer Center and Research Institute Tampa FL USA; ^6^ Department of Tumor Biology H. Lee Moffitt Cancer Center and Research Institute Tampa FL USA; ^7^ Chemical Biology and Molecular Medicine Program H. Lee Moffitt Cancer Center and Research Institute Tampa FL USA; ^8^ Sarcoma Department H. Lee Moffitt Cancer Center and Research Institute Tampa FL USA

**Keywords:** genomics, immune checkpoint inhibitor therapy, Merkel cell carcinoma, Merkel cell polyomavirus, next‐generation sequencing

## Abstract

**Background:**

The use of targeted therapy remains a treatment consideration for some patients with advanced Merkel cell carcinoma (MCC). However, supportive data on the use of targeted therapy approaches are limited. Thus, we sought to evaluate the responsiveness of targeted agents in patients with advanced MCC.

**Methods:**

An institutional MCC database identified patients who were treated with targeted therapy. For the purpose of this study, targeted therapy was defined as any multi‐targeted tyrosine kinase inhibitor or inhibitor of the PI3K‐pathway. Clinical benefit was defined as complete response, partial response, or stable disease (SD) ≥6 months. A subset of patient samples underwent next‐generation sequencing (NGS), Merkel cell polyomavirus testing, and PD‐L1/PD‐1 expression testing.

**Results:**

Nineteen patients with MCC treated with targeted therapy were identified, 21 targeted therapy regimens were evaluable for response in 18 patients. Four of twenty‐one (19%) of evaluable regimens were associated with clinical benefit with the best overall response of SD. The durations of SD were 13.6 months (59 weeks), 9.7 months (42 weeks), 7.6 months (33 weeks), and 7.2 months (31 weeks). Of the four patients who derived clinical benefit, three were treated with pazopanib alone and one was treated with pazopanib plus everolimus. No difference in the rate of clinical benefit between molecular disease subtypes was detected nor was associated with any specific genomic alteration.

**Conclusion:**

In our series, targeted agents elicited a disease control rate of 19% in patients with advanced MCC, with a best overall response of SD. Pazopanib alone or in combination exhibited a rate of disease control of 36% (4 of 11 with SD ≥6 months).

## Graphical abstract

The use of targeted therapy remains a treatment consideration for some patients with advanced Merkel cell carcinoma (MCC); however, supportive data are limited. In this retrospective analysis, we find that treatment with pazopanib led to clinical benefit in 4/11 (36%) of patients with heavily pretreated advanced MCC. Outside of pazopanib, we were unable to find anecdotal evidence of any meaningful clinical activity for several other kinase inhibitors, although the use was limited to small patient numbers for each.

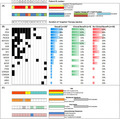

## 1 INTRODUCTION

Merkel cell carcinoma (MCC) is a rare and aggressive neuroendocrine malignancy of the skin. Previously, standard pharmacologic treatment for patients with MCC consisted of cytotoxic chemotherapy combinations, most commonly a platinum‐based agent plus etoposide.^1^, ^2^, ^3^, ^4^, ^5^ More recently, immune checkpoint inhibitors (ICI) have transformed the treatment of advanced MCC and are now standard frontline pharmacologic treatment.^6^, ^7^, ^8^, ^9^, ^10^ The use of ICI in the frontline setting is characterized by excellent response rates, ranging from 56% to 71%,^7^, ^11^, ^12^ in addition to frequent durable responses, with an estimated 74% of responses of ≥1 year in duration in one study.^9^ However, ICI do not universally induce responses and many patients do eventually experience disease progression, thus an unmet need remains. Further, some patients may not be candidates for immunotherapy, such as those who are immunosuppressed or have received organ transplant,^9^ thus need additional therapy options.

Beyond the demonstration of improved response rate and duration from ICI,^7^, ^9^, ^11^, ^12^ and the limited duration of benefit (<8 months) from cytotoxic chemotherapy,^5^ the efficacy of other pharmacologic approaches for MCC has not been well established. Tyrosine kinase inhibitor (TKI) therapy, while mainstream for many cancer types, to date, has been subject to limited investigation in MCC. A biological basis for the use of TKIs for the treatment of patients with MCC is provided by the frequent expression of vascular endothelial growth factors, platelet‐derived growth factor B, and C‐kit, in addition to mutations in receptor tyrosine kinases and RAF‐family members.^13^, ^14^, ^15^, ^16^ Several case reports have described beneficial responses to a range of TKIs such as pazopanib, cabozantinib, imatinib, and idelalisib.^16^, ^17^, ^18^, ^19^ Early phase trials utilizing these agents have produced mixed results. A phase II trial of imatinib in 23 patients with metastatic or unresectable MCC reported a median overall survival of 5 months, including a partial response (PR) in one patient and a prolonged stable disease (SD) in another.^20^ A phase II study of pazopanib in patients with metastatic MCC reported clinical benefit in 56% (9/16) patients, with PR in 19% (3/16).^21^ A phase II study of patients with recurrent/metastatic MCC treated with cabozantinib was prematurely stopped after eight patients enrolled due to futility and toxicity. One patient had SD for 8 months.^22^

Nonetheless, on the basis of these limited published clinical data, targeted therapies remain a treatment consideration in certain circumstances when alternatives are lacking. It has been suggested that clinical benefits from these agents should include SD given the expectation of rapid progression in untreated advanced MCC and that future studies should incorporate predictive biomarkers to help patient selection.^16^ Advances in genomic technology and the more widespread incorporation of next‐generation sequencing (NGS) into the clinic have made evaluations of potential biomarkers for targeted therapy response more feasible. Recent studies have described two major subgroups of MCC, those which demonstrate a UV‐mutational signature with high tumor mutational burden (TMB) and those with few mutations and typically express the Merkel cell polyomavirus (MCPyV).^23^, ^24^, ^25^, ^26^, ^27^, ^28^, ^29^ Despite these clear differences in mutational profile, the two subgroups of MCC are indistinguishable in both their clinical presentation and in their response to immunotherapy.^8^, ^11^, ^23^, ^30^

Thus, we sought to evaluate the responsiveness of targeted agents in patients with advanced MCC and describe any association between response and specific genomic alterations in patients who underwent NGS as part of clinical care.

## 2 SUBJECTS, MATERIALS, AND METHODS

### 2.1 Study population

A retrospective review identified patients diagnosed with MCC who were treated at a single academic cancer center. Patients with MCC who received treatment with targeted therapy were identified using an institutional MCC registry^31^ and a database of all patients who underwent NGS as part of clinical care.^23^

### 2.2 Data collection

A retrospective chart review collected demographic, clinical, disease, treatment, genomic, and outcome variables on all individuals who were diagnosed with MCC and received treatment with targeted therapy. Targeted therapy was defined as any multi‐targeted TKI or inhibitor of the PI3K‐pathway. Multi‐targeted kinase inhibitors such as pazopanib and imatinib have been previously evaluated in clinical trials of patients with advanced MCC. In addition to pazopanib and imatinib, case reports have also described responses in patients treated with cabozantinib and inhibitors of the PI3K pathway.

### 2.3 Clinical review

Patients were deemed to derive clinical benefit if, at any point during treatment with a targeted agent, they were assessed as having a complete response (CR), PR, or SD for at least 6 months. The inclusion of SD as part of clinical benefit is in line with a previous suggestion of SD as a meaningful endpoint in patients with MCC treated with targeted therapies.^16^ Response assessments were determined by the retrospective review of clinic notes and imaging studies that were performed as part of routine clinical care, following RECIST 1.1 criteria.

### 2.4 Genomic analysis, viral detection, and PD‐L1/PD‐1 expression testing

Patient charts were reviewed to identify those that had undergone NGS of tumor tissue as part of clinical care. NGS consisted of genomic profiling of 322 unique cancer‐related genes, including the evaluation of TMB and mutational signatures performed in a Clinical Laboratory Improvement Amendments (CLIA)‐certified, College of American Pathologists (CAP)‐accredited, New York State approved laboratory (Foundation Medicine) as described previously.^23^, ^32^, ^33^ All cases that had undergone NGS also underwent additional analyses for the detection of the MCPyV and expression of PD‐L1 and PD‐1 as described previously.^23^

Viral analysis was conducted by NGS as well as immunohistochemistry (IHC). Detection of DNA sequences consistent with genomic MCPyV DNA by NGS was made through Velvet de novo assembly of off‐target sequencing reads left unmapped to the human reference genome (hg19). Detection by IHC for viral antigens was performed using the CM2B4 and MCV203Ab3 antibodies. Both methods have been described previously.^23^

All patients whose tumors had NGS performed were classified as either UV‐driven or viral driven based on their molecular profile and the detection of viral genomic DNA.

### 2.5 Statistical analysis

The primary endpoint was the rate of clinical benefit, defined as CR, PR, or SD for at least 6 months as determined by the treating physician. Secondary endpoints included the rate of clinical benefit based on specific treatment regimens and within molecular subgroups of UV‐driven and viral‐driven MCC in addition to the duration of therapy in the overall cohort and within the subgroups. While the full analysis set comprised all patients who received at least one dose of any targeted therapy, the endpoints are described only within patients who were evaluable for response.

## 3 RESULTS

A total of 19 patients with advanced MCC who received treatment with targeted therapy were identified (Table 1). All identified patients were white males and the median age at diagnosis was 75 years (range 18–90). Most patients (95%; 18/19) were diagnosed with MCC at an advanced stage of III or greater. Of the 19 treated patients, three were treated with two separate targeted therapy regimens, thus 22 targeted therapy regimens were used. The targeted agents used include pazopanib (*n* = 12), everolimus (*n* = 5), lenvatinib (*n* = 2), sunitinib (*n* = 2), and imatinib (*n* = 2) either alone, in combination, or in sequence. One patient (5%) was first treated with targeted therapy in the first‐line, five patients (26%) in the second‐line, six patients (32%) in the third line, and the remaining seven patients (37%) first received targeted therapy in the fourth‐line or later. All patients received treatment with cytotoxic chemotherapy and most (74%; 14/19) were treated with chemotherapy prior to their first treatment with targeted therapy. The majority of patients (89%; 17/19) were also treated with an ICI during the course of their treatment, most of these (71%; 12/17) were treated prior to targeted therapy. Notably, only 8% (1/12) of these cases achieved an objective response to treatment with an ICI.

**TABLE 1:** Baseline characteristics and treatment data for patients included

Characteristic	Value
**Baseline characteristics (*n* = 19)**	
Age (years)	
At diagnosis, median (range, *n* = 19)	75 (18–90)
At sequencing biopsy, median (range, *n* = 14	73 (18–86)
At sequencing, median (range, *n* = 14)	74 (19–87)
Sex	
Male	19 (100%)
Female	0 (0%)
Race	
White	19 (100%)
Ethnicity	
Non‐Hispanic	19 (100%)
Stage at the time when sequencing was performed (*n* = 14)	
IIIB	2 (14%)
IV	12 (86%)
Tumor utilized for sequencing (*n* = 14)	
Archival primary tumor	4 (29%)
Metastatic tumor	10 (71%)
**Treatment data (*n* = 19)**	
Targeted therapy agents used	
Pazopanib	12 (63%)
Everolimus	5 (26%)
Lenvatinib	2 (11%)
Sunitinib	2 (11%)
Imatinib	2 (11%)
Targeted therapy regimens used	
Pazopanib	10 (53%)
Everolimus	3 (16%)
Imatinib	2 (11%)
Sunitinib; pazopanib	1 (5%)
Sunitinib; everolimus	1 (5%)
Lenvatinib	1 (5%)
Lenvatinib; pazopanib + everolimus	1 (5%)
Line of first targeted therapy use	
1	1 (5%)
2	5 (26%)
3	6 (32%)
4	3 (16%)
5+	4 (21%)
Immunotherapy treated	
Yes	17 (89%)
No	2 (11%)
Relationship to immunotherapy (*n* = 17)	
Immunotherapy first	12 (71%)
Targeted therapy first	5 (29%)
Relationship to chemotherapy	
Chemotherapy first	14 (74%)
Targeted therapy first	5 (26%)

Note

Data are *n* (%) unless stated otherwise.

**FIGURE 1:**
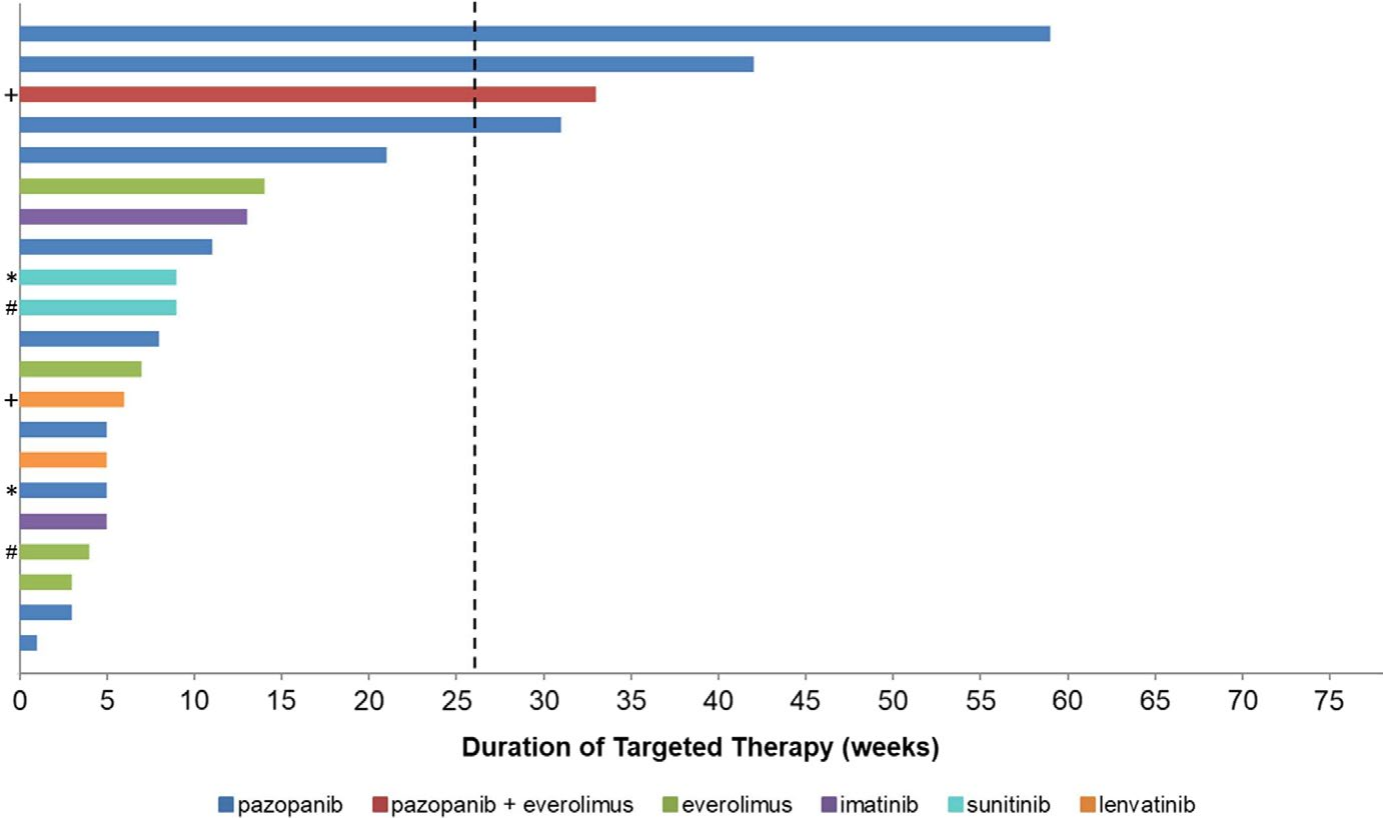
Duration of targeted therapy. Matched symbols (+, *, #) indicate multiple lines of treatment for the same patient. Vertical dashed line at 26 weeks indicates the threshold for the determination of clinical benefit. Only treatment duration for evaluable patients are shown

Of the 22 targeted therapy treatments used, 21 were evaluable for response in 18 patients. One patient was not evaluable for response as treatment was switched to ICI when the drug became available after 4 weeks of treatment with pazopanib without toxicity or progression and thus was excluded from response analysis. Nineteen percent (4/21) of targeted therapy regimens were associated with clinical benefit with a best overall response of SD (0 PR and 4 SD > 26 weeks) (Figure 1; Table 2). The median duration of treatment was 8 weeks (range = 3–59). The durations of clinical benefit were 13.6 months (59 weeks), 9.7 months (42 weeks), 7.6 months (33 weeks), and 7.2 months (31 weeks). Of the four patients who derived clinical benefit, three were treated with pazopanib alone and one was treated with pazopanib plus everolimus. Thus, 4 of 11 (36%) evaluable patients treated with pazopanib derived clinical benefit.

**TABLE 2:** Response data by treatment regimen

Response	Pazopanib (*n* = 10)	Everolimus (*n* = 4)	Sunitinib (*n* = 2)	Lenvatinib (*n* = 2)	Imatinib (*n* = 2)	Pazopanib + everolimus (*n* = 1)	All (*n* = 21)
Overall response rate	0	0	0	0	0	0	0
Best overall response							
Complete response	0	0	0	0	0	0	0
Partial response	0	0	0	0	0	0	0
Stable disease	3 (30%)	0	0	0	0	1 (100%)	4 (19%)
Progressive disease	7 (70%)	4 (100%)	2 (100%)	2 (100%)	2 (100%)	0	17 (81%)
Disease control rate	3 (30%)	0	0	0	0	1 (100%)	4 (19%)
mDOT (weeks)	9.5	6	9	5.5	9	33	8
DOT range (weeks)	1–59	3–14	9	5–6	5–13	33	1–59

Note

Data represent the best overall response by treatment regimen received. Data shown are from patients who were evaluable for response.

Abbreviation: mDOT, median duration of therapy.

Overall response rate and Disease control rate are the most important rows and summarize the rows below them (in highlighted).

**FIGURE 2:**
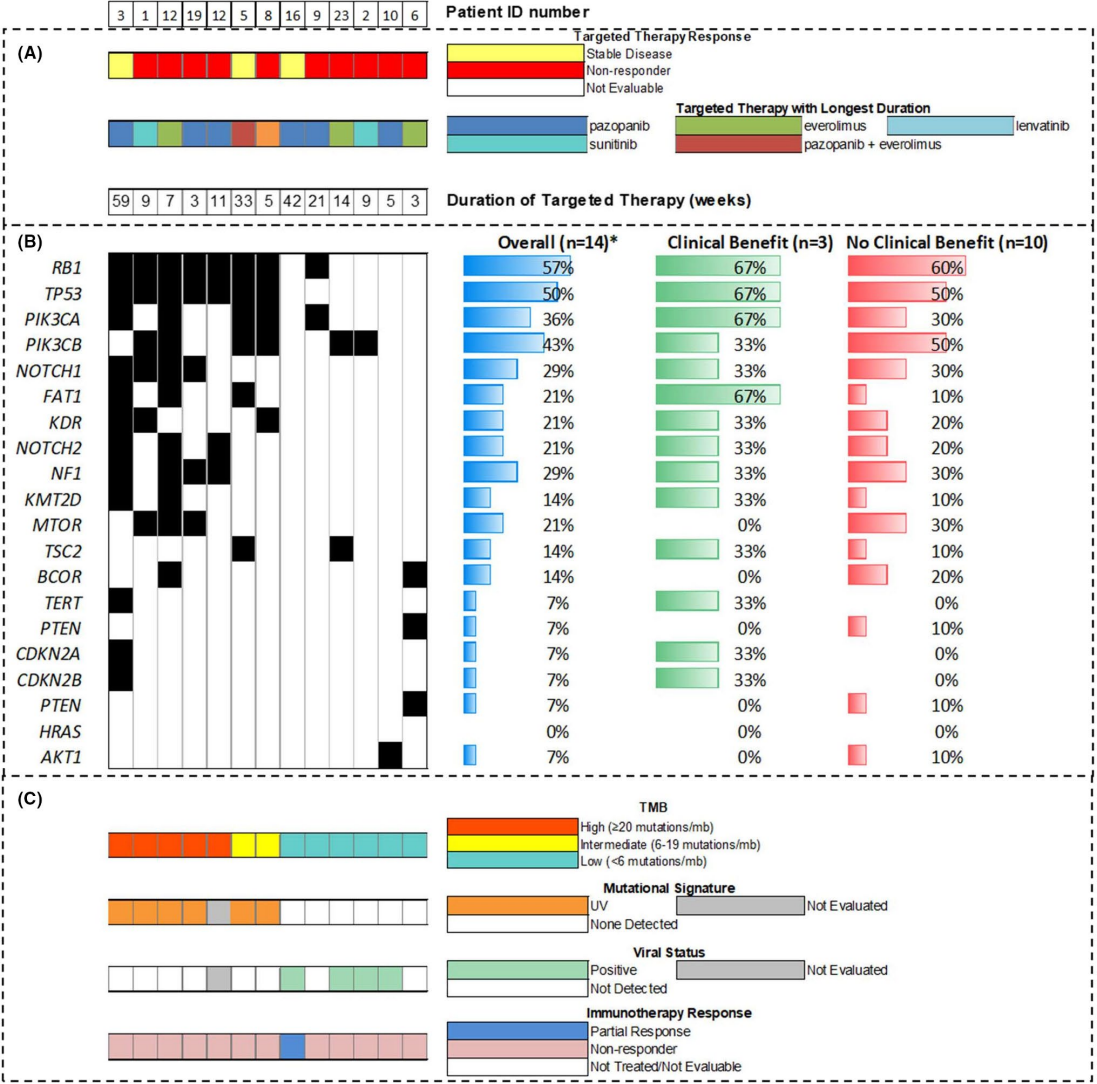
Genomic correlates of response. Fourteen patients within the cohort who underwent nextgenerations sequencing (NGS) as part of clinical care. Each patient is represented within an individual column. A, Targeted therapy treatment. B, Oncoprint and association between mutation and response. The percentages represent the percentage of patients in each column (overall/clinical benefit/no clinical benefit) with a mutation in the gene in that row. For example, in row 1 (RB1) 7/14 (50%) patients had a mutation in RB1, 2/3 (67%) with clinical benefit had a mutation in RB1, and 6/10 (60%) of patients with no clinical benefit had a mutation in RB1, and so forth. *One patient was not evaluable for response as treatment was switched to ICI when drug became available after four weeks of treatment with pazopanib without toxicity or progression. C, Other clinical/treatment variables

Fourteen (74%) patients had NGS performed on their tumors (Figure 2). Responses to targeted therapy were evaluable in 13 of these patients, 3 (23%) of whom derived clinical benefit. The tumor sample from one pazopanib‐responsive patient had alterations in *FGFR1*, *KIT*, and *KDR*, while the other had no alterations in genes associated with pazopanib activity. The tumor sample from the pazopanib plus everolimus‐responsive patient had a *PIK3CA* A1035V alteration not known to be activating.

Twelve of the patients whose tumors had NGS performed were also classified as UV‐driven versus viral driven based on their molecular profile and the detection of viral genomic DNA. Clinical benefit rates were 33% (2/6) in the UV‐driven group and 17% (1/6) in the viral‐driven group.

Given the clinical benefit rate observed specifically in patients treated with pazopanib, we evaluated clinical and molecular parameters as potential correlates of response specifically in this subgroup of patients (Table 3). We observed a notable trend in patients deriving benefit being more likely to be previously treated with ICI (100% vs. 57.1%, *p* = 0.24) and more likely to have a matched genomic finding that would suggest benefit (67% vs. 0%, *p* = 0.11); however, the small number of patients in each group precluded meaningful statistical comparisons.

**TABLE 3:** Comparison of pazopanib‐treated patients

Clinical benefit from pazopanib	Clinical benefit from pazopanib		*p*
	Yes (*n* = 4)	No (*n* = 7)	
Prior immune checkpoint inhibitor therapy	4 of 4 (100%)	4 of 7 (57%)	0.24
>2 previous lines of therapy	4 of 4 (100%)	3 of 7 (43%)	0.19
Viral molecular subtype (if NGS available)	1 of 3 (33%)	3 of 5 (60%)	1
Matched genomic finding (if NGS available)	2 of 3 (67%)	0 of 5 (0%)	0.11

Note

Viral molecular subtype and NGS data were available in three of the four patients who derived clinical benefit from pazopanib and five of the seven patients who did not, thus that analysis was limited to those three and five patients.

Abbreviation: NGS, next‐generation sequencing.

## 4 DISCUSSION

In this study, we report our institutional treatment outcomes of patients with advanced MCC treated with targeted therapy. This inquery addresses an area of unmet clinical need as treatment with targeted therapy remains a treatment consideration for some patients, particularly those who are relapsed/refractory to ICI or cytotoxic chemotherapy and those ineligible for treatment with ICI. In our cohort, we observed clinical benefit exclusively in patients treated with pazopanib, which was associated with a clinical benefit rate of 36% (4/11).

It is a challenge to compare the results reported here with other reports due to a general paucity of data on the clinical outcomes of patients with advanced MCC treated with targeted therapies. However, these findings are consistent with the results of a phase II study of pazopanib that reported clinical benefit in 56% (9/16) of patients. Notable, however, is that PRs were reported in 19% (3/16) of patients on the phase II trial, compared with none in our series.^21^ Notably, the patients included in our analysis were heavily pre‐treated. The majority received treatment with chemotherapy or immunotherapy prior to treatment with targeted therapy, and 68% of patients received targeted therapy in the third line or later.

The identification of predictors of benefit from systemic therapies remains elusive in MCC. When evaluated across our entire cohort with various TKI treatments, there was no apparent difference in the rate of clinical benefit between MCC molecular subtypes nor with molecular features. When evaluating clinical benefit from pazopanib specifically, we did observe an interesting trend toward benefiting patients being more heavily pretreated, more likely to be treated with prior ICI, and more likely to have a matched genomic feature that might suggest a response. However, all of these comparisons were between small patient numbers and would need validation across a greater sample size to draw meaningful conclusions. Further, in regard to the molecular correlates to response, the driving nature of the specific molecular findings identified in our cohort would require further validation. One of the three sequenced patients who derived clinical benefit from pazopanib had alterations in three genes associated with its tyrosine kinase activity (*FGFR1*, *KDR*, *KIT*). However, none of the specific alterations observed have been previously definitively characterized as activating. Thus, it is uncertain which of these if any are clear driver mutations that could be directly inhibited by pazopanib. Similarly, while the patient who derived clinical benefit from treatment with pazopanib plus everolimus had a *PIK3CA* A1035V mutation, this alteration has also not been conclusively established as activating or sensitive to pathway inhibitors. Genomic findings might ultimately help to select appropriate patients for pazopanib therapy to better enrich those likely to benefit; however, this approach would require further validation. It is also notable that at least one patient that underwent NGS testing and had no matched genomic findings achieved significant clinical benefit from pazopanib.

This study has limitations. Notably, it is difficult to draw meaningful conclusions from a 19‐patient study. Further, the pre‐treatment conditions were quite variable among our cohort, making conclusions regarding efficacy even more challenging. However, given the rarity of MCC, it is noteworthy that this study is comparable in size to the largest prospective studies of targeted therapy in MCC of 23, 19, and 8 patients.^18^, ^19^, ^20^ Thus, this study represents a significant portion of the available data in the published literature.

For patients with advanced MCC refractory to, or ineligible for ICI therapy, treatment options likely to result in durable responses are limited. While ideally these patients should be treated on the clinical trial, trials are not always readily available or patients may not be a trial candidate. Given the rapidly progressive nature of advanced MCC, despite seeing no objective responses, we believe that our experience reported here demonstrates the benefit of pazopanib in a subset of patients as SD of >6 months is clinically meaningful in this disease setting. Therefore, for ICI refractory or ineligible patients without an available clinical trial option, we believe that the use of pazopanib is a reasonable treatment consideration given limited alternatives. Our results help to confirm those of a single small phase II study of pazopanib in this disease and perhaps give a more realistic set of efficacy expectations given the “real world” conditions and heavily pretreated nature of our treatment cohort.

## 5 CONCLUSION

In this retrospective analysis, we find that treatment with pazopanib led to clinical benefit in 4/11 (36%) of patients with heavily pretreated advanced MCC. Outside of pazopanib, we were unable to find anecdotal evidence of any meaningful clinical activity for several other kinase inhibitors, although the use was limited to small patient numbers for each.

## 6 ETHICS APPROVAL STATEMENT

This study was approved under IRB protocol #19191 (Pro00022458) at Moffitt Cancer Center.

## CONFLICT OF INTEREST

J.A.D. received research funding from Constellation Pharmaceuticals and has served as a consultant to Merck & Co. and EMD Serono. N.I.K. has consulting or advisory roles with BMS, Merck, Regeneron, Immunocore, Array, Jounce, Iovance, has received honoraria from Sanofi, is on a data safety monitoring board for AstraZeneca and Incyte, has received research funding (to institute) from BMS, Merck, Celgene, Novartis, HUYA, Regeneron, Replimune, and GSK, and owns common stock (self) in Amarin, TransEnterix, Bellicum, and Mazor Robotics. K.Y.T. has advisory board relationships with Nanosive and NFlection Therapeutics. A.S.B. has consulting or advisory roles with Bayer, Deciphera, and EMD Serono and his spouse have provided expert testimony for GlaxoSmithKline. The other authors report no conflicts of interest.

## AUTHOR CONTRIBUTION

Conception and design: TCK, KTY, and ASB. Development of methodology: TCK and ASB. Acquisition of data: All authors. Analysis and interpretation of data: TCK and ASB. Writing, review, and/or revision of the manuscript: All authors. Study supervision: KYT and ASB.

## Data Availability

The data that support the findings of this study are available on request from the corresponding author. The data are not publicly available due to privacy or ethical restrictions.

## References

[1] Sharma D, Flora G, Grunberg SM. Chemotherapy of metastatic Merkel cell carcinoma: case report and review of the literature. Am J Clin Oncol. 1991;14(2):166‐169. 2028925 10.1097/00000421-199104000-00014

[2] Voog E, Biron P, Martin JP, Blay JY. Chemotherapy for patients with locally advanced or metastatic Merkel cell carcinoma. Cancer. 1999;85(12):2589‐2595. 10375107 10.1002/(sici)1097-0142(19990615)85:12<2589::aid-cncr15>3.0.co;2-f

[3] Tai PT, Yu E, Winquist E, et al. Chemotherapy in neuroendocrine/Merkel cell carcinoma of the skin: case series and review of 204 cases. J Clin Oncol. 2000;18(12):2493‐2499. 10856110 10.1200/JCO.2000.18.12.2493

[4] Iyer JG, Blom A, Doumani R, et al. Response rates and durability of chemotherapy among 62 patients with metastatic Merkel cell carcinoma. Cancer Med. 2016;5(9):2294‐2301. 27431483 10.1002/cam4.815 PMC5055152

[5] Nghiem P, Kaufman HL, Bharmal M, Mahnke L, Phatak H, Becker JC. Systematic literature review of efficacy, safety and tolerability outcomes of chemotherapy regimens in patients with metastatic Merkel cell carcinoma. Future Oncol. 2017;13(14):1263‐1279. 28350180 10.2217/fon-2017-0072 PMC6040046

[6] Bradford D, Demko S, Jin S, et al. FDA accelerated approval of pembrolizumab for recurrent locally advanced or metastatic Merkel cell carcinoma. Oncologist. 2020;25(7):e1077‐e1082. 32272501 10.1634/theoncologist.2020-0184 PMC7356706

[7] D'Angelo SP, Bhatia S, Brohl AS, et al. Avelumab in patients with previously treated metastatic Merkel cell carcinoma: long‐term data and biomarker analyses from the single‐arm phase 2 JAVELIN Merkel 200 trial. J Immunother Cancer. 2020;8(1):e000674. 32414862 10.1136/jitc-2020-000674 PMC7239697

[8] Kaufman HL, Russell J, Hamid O, et al. Avelumab in patients with chemotherapy‐refractory metastatic Merkel cell carcinoma: a multicentre, single‐group, open‐label, phase 2 trial. Lancet Oncol. 2016;17(10):1374‐1385. 27592805 10.1016/S1470-2045(16)30364-3 PMC5587154

[9] Kaufman HL, Russell JS, Hamid O, et al. Updated efficacy of avelumab in patients with previously treated metastatic Merkel cell carcinoma after >/=1 year of follow‐up: JAVELIN Merkel 200, a phase 2 clinical trial. J Immunother Cancer. 2018;6(1):7. 29347993 10.1186/s40425-017-0310-x PMC5774167

[10] Nghiem P, Bhatia S, Lipson EJ, et al. Durable tumor regression and overall survival in patients with advanced Merkel cell carcinoma receiving pembrolizumab as first‐line therapy. J Clin Oncol. 2019;37(9):693‐702. 30726175 10.1200/JCO.18.01896 PMC6424137

[11] Nghiem PT, Bhatia S, Lipson EJ, et al. PD‐1 blockade with pembrolizumab in advanced Merkel‐cell carcinoma. N Engl J Med. 2016;374(26):2542‐2552. 27093365 10.1056/NEJMoa1603702 PMC4927341

[12] Topalian SL, Bhatia S, Hollebecque A, et al. Abstract CT074: non‐comparative, open‐label, multiple cohort, phase 1/2 study to evaluate nivolumab (NIVO) in patients with virus‐associated tumors (CheckMate 358): efficacy and safety in Merkel cell carcinoma (MCC). Can Res. 2017;77(13 Supplement):CT074‐CT.

[13] Harms PW, Harms KL, Moore PS, et al. The biology and treatment of Merkel cell carcinoma: current understanding and research priorities. Nat Rev Clin Oncol. 2018;15(12):763‐776. 30287935 10.1038/s41571-018-0103-2 PMC6319370

[14] Brunner M, Thurnher D, Pammer J, et al. Expression of VEGF‐A/C, VEGF‐R2, PDGF‐alpha/beta, c‐kit, EGFR, Her‐2/Neu, Mcl‐1 and Bmi‐1 in Merkel cell carcinoma. Mod Pathol. 2008;21(7):876‐884. 18408656 10.1038/modpathol.2008.63

[15] Villani A, Fabbrocini G, Costa C, Carmela Annunziata M, Scalvenzi M. Merkel cell carcinoma: therapeutic update and emerging therapies. Dermatol Ther (Heidelb). 2019;9(2):209‐222. 30820877 10.1007/s13555-019-0288-z PMC6522614

[16] Tarabadkar ES, Thomas H, Blom A, et al. Clinical benefit from tyrosine kinase inhibitors in metastatic Merkel cell carcinoma: a case series of 5 patients. Am J Case Rep. 2018;19:505‐511. 29706615 10.12659/AJCR.908649 PMC5952731

[17] Loader DE, Feldmann R, Baumgartner M, et al. Clinical remission of Merkel cell carcinoma after treatment with imatinib. J Am Acad Dermatol. 2013;69(4):e181‐e183. 24034390 10.1016/j.jaad.2013.03.042

[18] Davids MS, Charlton A, Ng SS, et al. Response to a novel multitargeted tyrosine kinase inhibitor pazopanib in metastatic Merkel cell carcinoma. J Clin Oncol. 2009;27(26):e97‐e100. 19564526 10.1200/JCO.2009.21.8149

[19] Shiver MB, Mahmoud F, Gao L. Response to idelalisib in a patient with stage IV Merkel‐cell carcinoma. N Engl J Med. 2015;373(16):1580‐1582. 10.1056/NEJMc1507446 PMC4652581 26466009

[20] Samlowski WE, Moon J, Tuthill RJ, et al. A phase II trial of imatinib mesylate in Merkel cell carcinoma (neuroendocrine carcinoma of the skin): a Southwest Oncology Group study (S0331). Am J Clin Oncol. 2010;33(5):495‐499. 20019577 10.1097/COC.0b013e3181b9cf04 PMC2978644

[21] Nathan PD, Gaunt P, Wheatley K, et al. UKMCC‐01: A phase II study of pazopanib (PAZ) in metastatic Merkel cell carcinoma. J Clin Oncol. 2016;34(15):9542.

[22] Rabinowits G, Lezcano C, Catalano PJ, et al. Cabozantinib in patients with advanced Merkel cell carcinoma. Oncologist. 2018;23(7):814‐821. 29445030 10.1634/theoncologist.2017-0552 PMC6058327

[23] Knepper TC, Montesion M, Russell JS, et al. The genomic landscape of Merkel cell carcinoma and clinicogenomic biomarkers of response to immune checkpoint inhibitor therapy. Clin Cancer Res. 2019;25(19):5961‐5971. 31399473 10.1158/1078-0432.CCR-18-4159 PMC6774882

[24] Moshiri AS, Doumani R, Yelistratova L, et al. Polyomavirus‐negative Merkel cell carcinoma: a more aggressive subtype based on analysis of 282 cases using multimodal tumor virus detection. J Invest Dermatol. 2017;137(4):819‐827. 27815175 10.1016/j.jid.2016.10.028 PMC5565758

[25] Harms PW, Vats P, Verhaegen ME, et al. The distinctive mutational spectra of polyomavirus‐negative Merkel cell carcinoma. Cancer Res. 2015;75(18):3720‐3727. 26238782 10.1158/0008-5472.CAN-15-0702 PMC4573907

[26] Wong SQ, Waldeck K, Vergara IA, et al. UV‐associated mutations underlie the etiology of MCV‐negative Merkel cell carcinomas. Cancer Res. 2015;75(24):5228‐5234. 26627015 10.1158/0008-5472.CAN-15-1877

[27] Goh G, Walradt T, Markarov V, et al. Mutational landscape of MCPyV‐positive and MCPyV‐negative Merkel cell carcinomas with implications for immunotherapy. Oncotarget. 2016;7(3):3403‐3415. 26655088 10.18632/oncotarget.6494 PMC4823115

[28] Gonzalez‐Vela MDC, Curiel‐Olmo S, Derdak S, et al. Shared oncogenic pathways implicated in both virus‐positive and UV‐induced Merkel cell carcinomas. J Invest Dermatol. 2017;137(1):197‐206. 27592799 10.1016/j.jid.2016.08.015

[29] Carter MD, Gaston D, Huang WY, et al. Genetic profiles of different subsets of Merkel cell carcinoma show links between combined and pure MCPyV‐negative tumors. Hum Pathol. 2018;71:117‐125. 29079179 10.1016/j.humpath.2017.10.014

[30] Schrama D, Peitsch WK, Zapatka M, et al. Merkel cell polyomavirus status is not associated with clinical course of Merkel cell carcinoma. J Invest Dermatol. 2011;131(8):1631‐1638. 21562568 10.1038/jid.2011.115

[31] Cohen L, Muradova E, Knepper T, Brohl A, Tsai K. merkel cell carcinoma: the Moffitt Cancer Center experience: 9769. J Am Acad Dermatol. 2019;81(4):AB239.

[32] Frampton GM, Fichtenholtz A, Otto GA, et al. Development and validation of a clinical cancer genomic profiling test based on massively parallel DNA sequencing. Nat Biotechnol. 2013;31(11):1023‐1031. 24142049 10.1038/nbt.2696 PMC5710001

[33] Sun JX, He Y, Sanford E, et al. A computational approach to distinguish somatic vs. germline origin of genomic alterations from deep sequencing of cancer specimens without a matched normal. PLoS Comput Biol. 2018;14(2):e1005965. 29415044 10.1371/journal.pcbi.1005965 PMC5832436

